# Multiple ectopic hepatocellular carcinomas in the pancreas: A case report

**DOI:** 10.1097/MD.0000000000006747

**Published:** 2017-07-28

**Authors:** Zhigui Li, Xiaoting Wu, Tianfu Wen, Chuan Li, Wen Peng

**Affiliations:** Department of General Surgery, West China Hospital, Sichuan University, Chengdu, China.

**Keywords:** ectopic hepatocellular carcinomas, ectopic liver, pancreas

## Abstract

**RATIONALE:**

Ectopic liver tissue can develop at various sites near the liver. Ectopic hepatocellular carcinomas (HCCs) arising from ectopic liver tissue have a rare clinical incidence. A very rare case has been observed to have metastasis after operation.

**PATIENT CONCERNS:**

We report an extremely rare case with multiple masses which were identified in the head and body of the pancreas.

**DIAGNOSES:**

Ectopic hepatocellular carcinomas.

**INTERVENTIONS:**

The masses were removed by surgical resection. Histopathological analysis showed that both masses were ectopic HCC.

**OUTCOMES:**

The patient was still alive and did not have metastasis and relapse.

**LESSONS:**

The literature review for this rare case is also presented to highlight the risk of ectopic HCC and good prognosis of operation for ectopic HCC.

## 1 Introduction

Ectopic liver tissue can develop at various sites near the liver, such as the abdominal cavity, thorax, gallbladder, and retroperitoneal cavity.^[1–4]^ As a very rare entity with an incidence between 0.24% and 0.47%,^[5]^ the presence of ectopic liver tissue is usually asymptomatic, but occasionally, it causes unexpected problems such as hepatocarcinogenesis.^[6]^ Therefore, hepatocellular carcinomas (HCCs) arising from ectopic liver tissue are even rarer.^[7]^ This report presents a case of a patient with multiple masses in the pancreas, the final diagnosis was of ectopic HCC.

## 2 Case report

**Figure 1:**
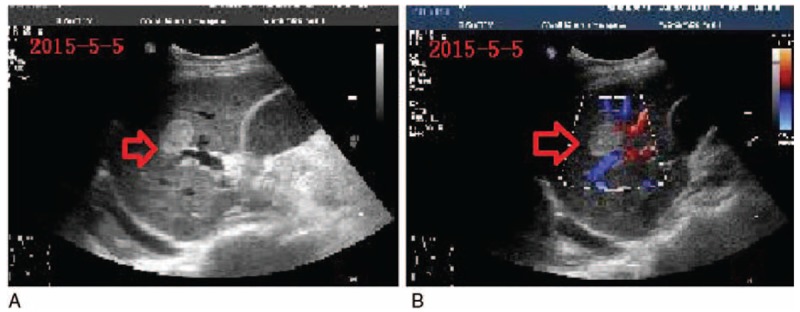
Ultrasonography showing multiple masses in the liver (red arrow). (A) A two-dimensional ultrasound. (B) Color Doppler ultrasound.

**Figure 2:**
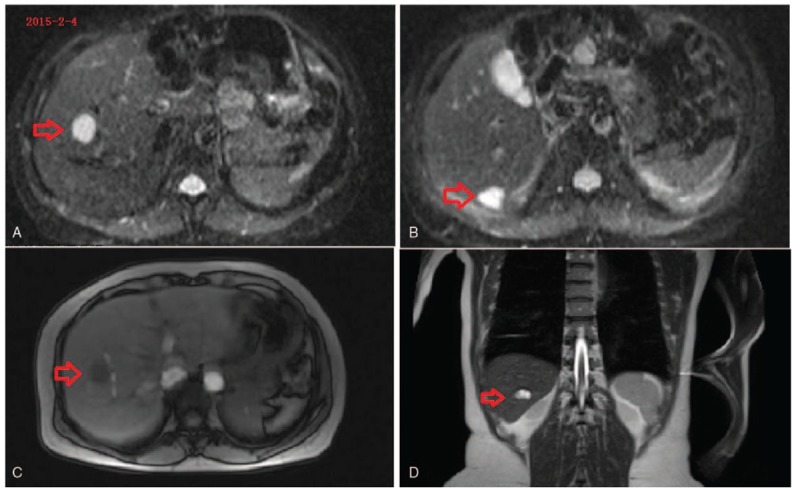
Contrast-enhanced magnetic resonance imaging showing multiple masses in the liver (red arrow). (A) Hemangioma in right anterior lobe; (B) hemangioma in right posterior lobe; (C) hemangioma in right anterior lobe; (D) hemangioma in right posterior lobe.

**Figure 3:**
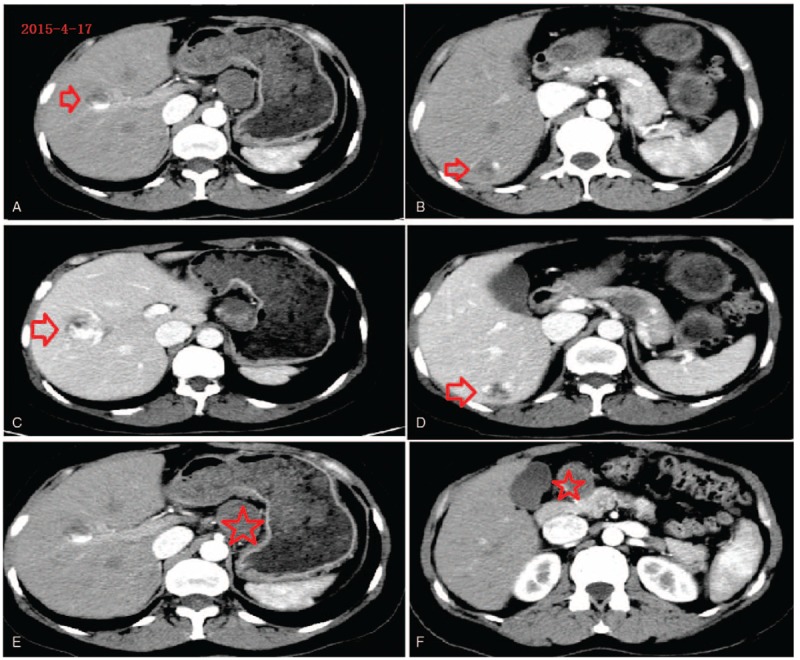
Contrast-enhanced computer tomography showing multiple masses in theliver (red arrow) and multiple masses in the pancreas (rea star). (A) Hemangioma in right anterior lobe, arterial phase; (B) hemangioma in right posterior lobe, arterial phase; (C) hemangioma in right anterior lobe,portal venous phase; (D) hemangioma in right posterior lobe,portal venous phase; (E) a mass in the superior body of the pancreas; (F) a mass in the superior head of the pancreas.

A 44-year-old female patient, who was nondiabetic and nonhypertensive, presented with an elevated serum alpha-fetoprotein (AFP) level and abdominal masses in the pancreas identified at a general health checkup in January 2015. Laboratory tests in January 2015 had revealed that her serum AFP level was higher than 1200 ng/mL (normal range: 0–8 ng/mL). She had taken Chinese traditional medicine from March 2015. The detailed ingredients of the Chinese medicine are not known. The patient was not a smoker, and did not consume excessive amounts of alcohol. She was known to have had a history of pulmonary tuberculosis 7 years ago, but had no recurrence until now. She had undergone total hysterectomy 2 years ago. On admission in April 2015, there were no symptoms of other conditions, such as fever, pain, diarrhea, or appetite loss with nausea and vomiting. We detected no significant changes in her feeding habits. She liked Chinese food and her intake amount was normal. Examination revealed that her abdomen was soft, with epigastric deep dull pain and without palpable masses. Her liver function tests were within normal limits. These tests include albumin, alanine aminotransferase (ALT), aspartate transaminase (AST), bilirubin (direct and indirect), prothrombin time, and so on. Hepatitis B core antibody (HBcAb) was positive, whereas other antibodies, including hepatitis B surface antibody (HBsAb), hepatitis B surface antigen (HBsAg), hepatitis B e antibody (HBeAb), hepatitis B e antigen (HBeAg), and hepatitis C antibody (anti-HCV) were negative. Her serum AFP level was 553.90 ng/mL. Tumor marker tests showed that carcinoembryonic antigen (CEA), human chorionic gonadotropin (HCG), and CA19-9 and CA12-5 were normal (data not shown). Ultrasonography (Fig. 1A, B), magnetic resonance imaging (Fig. 2A–D), and computer tomography (Fig. 3A–E) showed multiple hemangiomas of the liver, and multiple oval, solid, fixed masses adjacent to the pancreas. The masses measured 5 × 4 and 4 × 3 cm, respectively.

**Figure 4:**
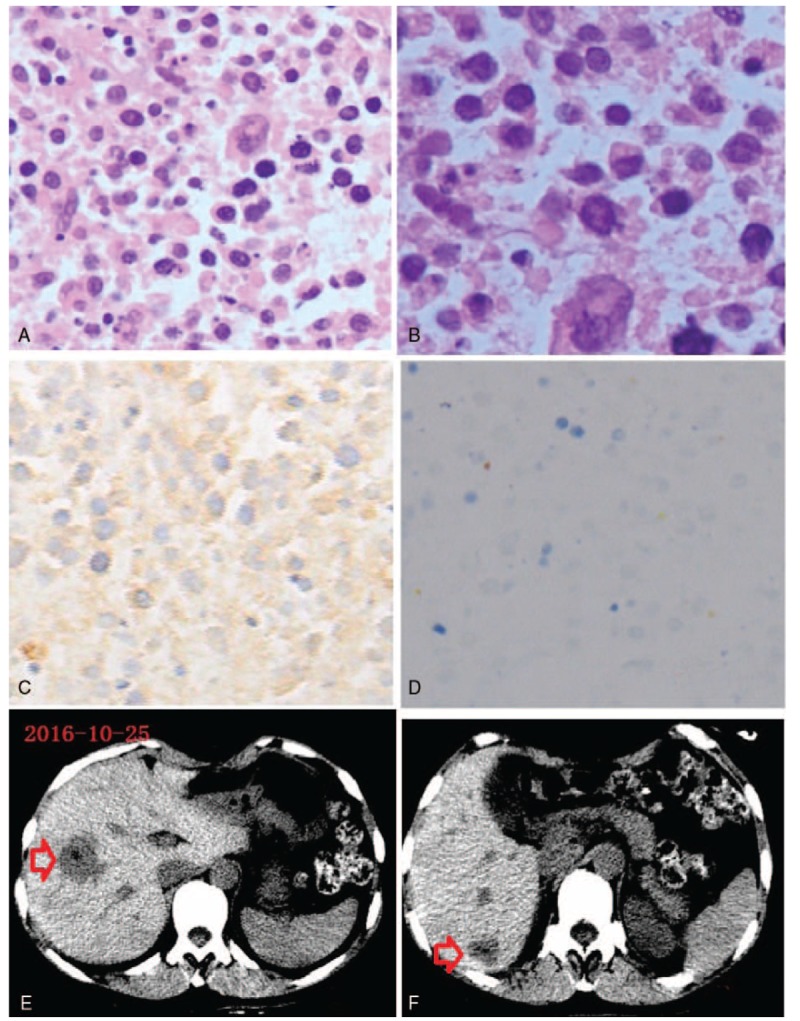
(A) Hematoxylin-eosin staining of a tumor section, ×200. (B) Hematoxylin-eosin staining of a tumor section, ×400. (C) Alpha-fetoprotein (AFP) staining of a tumor section, ×200; brown staining indicates AFP positive areas. (D) Chromogranin A (CgA) staining of a tumor section, ×200. (E) hemangioma in right anterior lobe (red arrow), plain scan; (F) hemangioma in right posterior lobe (red arrow), plain scan.

Concerning the high expense and nonspecificity of positron emission tomography-CT and the risk of tumor spread while endosonographically guided biopsy before surgery, the patient underwent open surgery under general anesthesia in May 2015. A transrectus incision was made, and tumors were exposed completely after resection of the colon ligament. During surgery, we found the liver was brownish red, and soft and multiple exogenous masses were attached separately to the body and head of the pancreas. Both masses were predominantly well circumscribed with separate thin capsules and they did not invade the pancreatic duct. The masses were located a few centimeters apart and were removed one by one. Little pancreatic tissue on the surface was removed. Microscopic examination of the specimen showed that the center of the tumors was necrotic and tumor cells had large ovoid nuclei (Fig. 4A, B). Immunohistochemical staining (Fig. 4C, D) of the tumor cells revealed that they were positive for cytokeratin (Pan) and AFP, Ki-67 (+80%), negative for cytokeratin 7, cytokeratin 20, Chromogranin A (CgA), synaptophysin (Syn), CEA, and Hep-Par-1. The final pathological diagnosis was HCC arising from ectopic liver tissue. Somatostatin was used to suppress pancreatic secretion for 3 days after surgery. The patient had a slight pancreatic leakage during postoperative recovery and the drainage tube remained for 1 month. Laboratory tests in August 2015 and October 2016 showed that serum AFP level was normal (data not shown). There was no significant change in body weight and glycosemia. The patient had experienced no recurrence at the last follow-up in October 2016.

The patient had provided the consent to publish the case report, and the consent procedure was approved by the Ethics Committee of West China Hospital of Sichuan University.

## 3 Discussion

Hepatic diverticulum, which can develop to liver, is derived from endothelial cell proliferation in the terminal ventral wall of foregut. Extrahepatic liver tissue can be classified into 2 major subgroups: ectopic liver, which is not connected to the main liver tissue, and accessory liver lobe, which is connected to the main liver tissue. At present, there are 3 main viewpoints of ectopic liver formation: first, the sites such as gallbladder, parahepatic ligament, diaphragm residued the original hepatocyte in the processing of embryonic development, or hepatocyte buds invaded into the thorax or diaphragm when diaphragmatic closure; second, the original hepatocytes migrated into the other sites and differentiated into liver tissue; third, the peduncular atrophy of accessory liver lobe led to loss a connection between accessory liver lobe and mother liver. This case had multiple lesions and had not found a connection with mother liver, so the first or second viewpoints ought to explain the reason.

Before the advances in imaging analysis, AFP was widely used as a tumor marker for the diagnosis of HCC^[8]^; in nearly 60% of cases, serum AFP is higher than 20 ng/mL, and recently, it has been considered to be a prognostic marker for HCC outcome.^[9]^ Increased serum AFP was evident in this patient 3 months before her examination in our clinic. We consider this to be an important sign of ectopic HCC. However, the AFP level had declined when she attended our clinic, although it remained above reference level. After full investigation of the HCC, we speculated that AFP levels may have declined because of tumorous necrosis. Currently, it remains unclear why this necrosis had occurred. There is a possibility that it is related to the self-administration of traditional herbal medicine, but we cannot evaluate this because we were unable to determine the medication taken. Alternatively, this necrosis may be natural disease progression due to a lack of a blood supply that resulted in necrosis.

In China, about 10% of population were infected with hepatitis B virus, one of the major risk factor for liver cirrhosis, which is a premalignant condition.^[10]^ Judging from the literature, more than 80% of patients with HCC had liver cirrhosis, but 6 of 22 ectopic HCC had mother liver cirrhosis.^[11]^ It seems that ectopic liver could be more prone to hepatocarcinogenesis rather than mother liver. This case report proved that the above theory in accordance with the patient without mother liver cirrhosis. We speculated that hepatocytes in ectopic liver tissue with poor blood supply, difficulty of biliary drainage, and those that behave like normal hepatocytes, which can metabolize and excrete bile so that ectopic liver tissues are subjected to specific additional carcinogenic factors, easily develop ectopic HCC. However, most reported cases of ectopic HCC in the pancreas are of single tumors.^[11–14]^ We describe here a case of ectopic HCC in a Chinese patient without other risk factors for HCC or liver disease, apart from occult HBV infection. This case shows that multiple independent HCC tumors are possible in the pancreas, so it is important not to rule out ectopic HCC when multiple masses with elevated serum AFP level are identified in the pancreas.

To my knowledge, contrast-enhanced CT scanning showed that HCCs were rapidly enhanced and rapidly subsided and hepatic hemangioma early had a high density of edge enhancement that gradually extended to the center; lasted for a long time; and delayed phase had density filling.^[15]^ A diagnosis of ectopic HCC necessarily requires the identification of noncancerous liver parenchyma. We firmly believed that the hepatic mass was hemangiomas rather than HCC for the following criteria: there is no history of liver cirrhosis and malignancy; the ultrasonography finding, contrast-enhanced CT scan, and contrast-enhanced MRI images were highly suspected in the diagnosis of hepatic hemangiomas; the lesions had no significant change at the over 1-year follow-up (Fig. 4E, F); and serum AFP level was normal after operation without the treatment of hepatic lesions. We did not think that the multiple mass in the pancreas is another diagnosis, including hepatoid adenocarcinomas and metastasis of HCC and so on (Table 1). Hepatoid tumors of the pancreas are a heterogeneous group including pure HCC, which also called for ectopic HCC and hepatoid adenocarcinomas commonly locating in the stomach or ovary. Hepatoid adenocarcinomas generally associated with a poor prognosis had morphological and immunohistochemical similarity to HCC and were mixed with adenocarcinomas. This case showed exclusively hepatocellular differentiation without acinar structure and was diagnosed as ectopic HCC by 2 independent experienced pathologists. Pathologist could easily distinguish pancreatic endocrine tumors from hepatoid tumors by morphology and immunochemistry. The pancreas occasionally contained other ectopic tissues such as ectopic spleen^[16]^ and ectopic pregnancy,^[17]^ but obviously the patient did not belong to this situation.

**Table 1:**
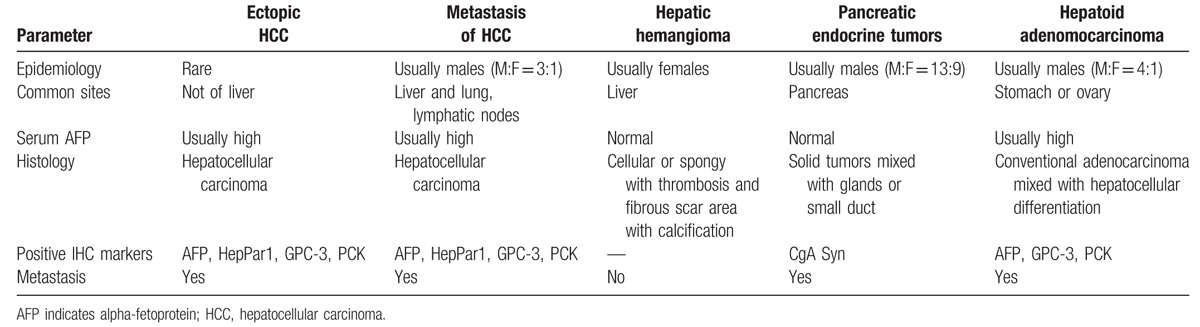
Differential diagnosis between ectopic HCC and other lesions.

The hemangioma is the disease of congenital vascular malformation that has a slight symptom and a high risk of rupture and thrombosis inside the tumor, especially for rapid increase in size and located on the edge. The patient is not candidate for surgical treatment on account of asymptomatic and small size and diffuse lesion of hemangioma and located centrally.

In this case, we found that both masses were circumscribed with a capsule during surgery, which were similar to some previous case reports.^[13,14]^ Because we considered that remaining pancreas tissue was important to prognosis, we decided to scoop out the multiple masses and reserve most of the pancreas tissue. It was really rare that the patients with ectopic HCC had metastasis before diagnosis.^[3,18]^ One case reported the patient with ectopic HCC died 15 months after the surgery,^[11]^ and we thought that the final diagnosis should be hepatoid adenocarcinomas in that pathologist found the lesion mixed with occasional acinar structures. The majority of HCCs in ectopic livers reported in Asian patients^[19]^ have not been observed of metastasis after operation and had a better prognosis so that the patient with clear resection margin did not necessarily receive chemotherapy. Until the article published, the patient was still alive and did not have metastasis and relapse. There were no significant changes in body weight and glycosemia, which indicated normal function of the pancreas. Therefore, we conclude that enucleation may be enough for early ectopic HCC.

In conclusion, we have reported a rare case of multiple ectopic HCC in pancreas presenting high AFP level. The tumors were simply scooped out and no relapse occurred till now. Enucleation may be enough for curative treatment of early ectopic HCC, even when multiple tumors are present.
